# Design and Comparative Evaluation of *In-vitro *Drug Release, Pharmacokinetics and Gamma Scintigraphic Analysis of Controlled Release Tablets Using Novel pH Sensitive Starch and Modified Starch- acrylate Graft Copolymer Matrices

**Published:** 2015

**Authors:** Pankaj Kumar, Ashok Laxmanrao Ganure, Bharat Bhushan Subudhi, Shubhanjali Shukla

**Affiliations:** a *School of Pharmaceutical Sciences, Siksha O Anusandhan University, Bhubaneswar, India.*; b *Sahyadri College of Pharmacy, Sangola, Solapur, India.*; c *Department of Pharmaceutics, Indian Institute of Technology (Banaras Hindu University), Varanasi, India.*

**Keywords:** Salbutamol sulphate, Methylmethacrylate, Graft copolymer, Acetylated starch, Korsmeyer’s model

## Abstract

The present investigation deals with the development of controlled release tablets of salbutamol sulphate using graft copolymers (St-g-PMMA and Ast-g-PMMA) of starch and acetylated starch. Drug excipient compatibility was spectroscopically analyzed via FT-IR, which confirmed no interaction between drug and other excipients. Formulations were evaluated for physical characteristics like hardness, friability, weight variations, drug release and drug content analysis which satisfies all the pharmacopoeial requirement of tablet dosage form. Release rate of a model drug from formulated matrix tablets were studied at two different pH namely 1.2 and 6.8, spectrophotometrically. Drug release from the tablets of graft copolymer matrices is profoundly pH-dependent and showed a reduced release rate under acidic conditions as compared to the alkaline conditions. Study of release mechanism by Korsmeyer’s model with n values between 0.61-0.67, proved that release was governed by both diffusion and erosion. In comparison to starch and acetylated starch matrix formulations, pharmacokinetic parameters of graft copolymers matrix formulations showed a significant decrease in C_max_ with an increase in t_max_, indicating the effect of dosage form would last for longer duration. The gastro intestinal transit behavior of the formulation was determined by gamma scintigraphy, using ^99m^Tc as a marker in healthy rabbits. The amount of radioactive tracer released from the labelled tablets was minimal when the tablets were in the stomach, whereas it increased as tablets reached to intestine. Thus, *in-vitro* and *in-vivo* drug release studies of starch-acrylate graft copolymers proved their controlled release behavior with preferential delivery into alkaline pH environment.

## Introduction

Controlled release technology has rapidly emerged over the past few decades as a new interdisciplinary science that offers novel approaches for delivery of bioactive agents into systemic circulation at a predetermined rate, achievement of optimum therapeutic responses, prolonged efficacy and decreased toxicity (1). Salbutamol sulphate (SS), a directly acting sympathomimetic drug, is a good candidate for controlled release formulations due to its short half-life (2-4 h) which necessitates frequent administration to maintain constant therapeutic levels but this becomes challenging because of its high water solubility (2). In recent years, use of natural polymers such as starch, cellulose, chitosan etc. as carriers in controlled drug delivery applications has attracted the attention of investigators because of their inherent biocompatibility, biodegradability and biosafety (3-5). But natural polymers also share some common disadvantages like poor flow properties, inadequate compression behavior, thermal liability and enormous swelling owing to their hydrophilic nature, which results in premature release of drug in the stomach/upper intestine, and therefore they should be protected while gaining entry into stomach and small intestine. This can be achieved by the modification of polysaccharides by various techniques like cross linking, addition of protective coating or grafting using acrylic monomers (6-8). Among the currently available graft copolymers, starch-based graft copolymers have fetched enormous attention due to their potential value as directly compressible excipients for controlled release matrices. Methyl methacrylate was chosen for grafting because of its known biocompatibility and non-toxic behavior, together with its hydrophobicity and ease of polymerization (9-10). Reservoir and matrix type tablets are the most commonly used controlled release preparations. Especially matrix tablets, which are prepared by direct compression technique, are one of the best formulation designs to sustain the release rate effectively over a period of 10 h; here in matrix system drug particles are homogenously embedded in the retardant polymeric material (11). Controlled release behavior of matrix system can be studied by gamma scintigraphy. Gamma scintigraphy is a preferred non-invasive imaging technique to determine the gastric residence time and transit time through the small intestine and colon (12).

In our previous research paper, we reported the synthesis and characterization of pH sensitive acetylated starch-acrylate graft copolymer (13) and to explore our previous research findings we directed our interest towards the design of oral controlled-release matrix tablets of salbutamol sulphate using starch based graft copolymers as hydrophobic inert matrix. Further, evaluations of tablets were planned for various physical characteristics, *in-vitro* dissolution, pharmacokinetics and gamma scintigraphic evaluation in rabbits upon oral administration. For optimized formulations, release kinetics and drug release mechanism were evaluated by fitting dissolution profile in various kinetic models *viz. *zero order, first order, Higuchi and Korsmeyer-Peppas models.

## Experimental

*Materials*

Maize starch was procured from Meru Chem Pvt. *Ltd*., India. *Hydroxypropyl Methylcellulose *K100M* (*HPMC K100M) was procured from Colorcon Asia Pvt. Ltd. and solvents of analytical grade were supplied by Merck *Ltd*., Germany. Gift sample of salbutamol sulphate was received from FDC *Ltd*., Mumbai, India. ^99m^Tc was provided as a gift sample from Spect Lab Nuclear Medicine Services, Pune, India.

*Synthesis of acetylated starch and graft copolymers [Starch grafted poly (methyl methacrylate) (St-g-PMMA) & Acetylated starch grafted poly (methyl methacrylate) (Ast-g-PMMA)]*

Acetylated starch and graft copolymers were synthesized and characterized by the methods previously described in our recent published paper (13). Here on starch back bone, methyl methacrylate was grafted via redox reaction. Wherein, Ce(IV) ion was reduced to Ce(III) ion and made an active site on starch back bone for grafting of methyl methacrylate. Synthesized samples were used for further study.

*Preparation of blends*

HPMC K100M polymer was chosen for comparison with graft copolymers for its controlled release properties. Various combinations were selected and blend was prepared for salbutamol sulphate using starch/acetylated starch/St-g-PMMA/Ast-g-PMMA/HPMC K100M, spay dried lactose and magnesium stearate. All ingredients were blended in Conta blender until a homogenous mixture was obtained with batch formula shown in Table 1. No more additives were included in order to get intrinsic information of the graft copolymeric material itself.

**Table 1:** Composition of salbutamol sulphate controlled release tablets

**S. No.**	**Ingredients**	**Formulation code**				
		**F1**	**F2**	**F3**	**F4**	**F5**
		**Qty (mg/tablets)**				
1	Salbutamol Sulphate	8	8	8	8	8
2	Maize Starch	159	---	---	---	---
3	Acetylated Starch (Ast)	---	159	---	---	---
4	St-g-MMA	---	---	159	---	---
5	Ast-g-MMA	---	---	---	159	---
6	Hypromellose (HPMC K 100M PREMIUM)	---	---	---	---	159
7	Spray dried lactose	30	30	30	30	30
8	Magnesium stearate	3	3	3	3	3
	Total Weight	200	200	200	200	200

*Preformulation study*

*Drug–excipient compatibility study by FT-IR spectroscopy*

FT-IR analysis was performed using physical mixture (1:1 w/w) of salbutamol sulphate with starch/acetylated starch/St-g-PMMA/Ast-g-PMMA.

*Angle of repose*

Angle of repose is an indicator of flowability of the material and it was determined by the fixed funnel and free standing cone method (14). In this method, blends of drug and excipients were poured through a funnel which was fixed at certain height (h) above the graph paper that was placed on a flat horizontal surface. Different blends were poured until the apex of conical pile just touched the tip of the funnel. Radius of the conical pile (r) was measured and the angle of repose (θ) was calculated by using below mentioned formula: 

θ= tan^-1^ (h/r)

*Formulation of tablets*

Tablets were prepared by direct compression method. The homogenous mixtures of different blends with suitable flow properties were shifted through #80 sieves, manually fed into the die and compressed in hydraulic press using a 8 mm flat faced beveled edge punch with the crushing force of 70-80 N. The average weight of tablets was 200 mg. Two hundred tablets of each batch were prepared and evaluated for various physicochemical parameters.

*Evaluation of tablets*

The physical testing on tablets of each batch was done after a relaxation period of at least 24 h. Weight variation test was performed on 20 individually weighed (Citizen CY204 Analytical Balance, Minnesota, USA) tablets according to the official method of United State Pharmacopoeia (USP/NF-32). The thickness and diameter of 10 tablets were measured individually using Vernier Caliper (Edutek instrumentation, Ambala, India) and an average value of thickness and diameter were calculated. The crushing strength (Kg/cm^2^) of prepared tablets was established using Monsanto hardness tester (MHT-20, Campbell Electronics, Mumbai, India). Tablets friability was determined by Roche friabilator (C-FT-20, Pharma Chem Machineries, Mumbai, India), and it was calculated as the percentage of weight loss in 20 tablets resulting from shock and attrition due to the revolution of plastic chamber operated for 4 minutes at 25 rpm.

*Drug content uniformity*

Ten tablets were weighed individually and crushed into a fine powder with a mortar and pestle. Crushed powder equivalent to 8 mg of the drug was weighed and transferred to a 50 mL volumetric flask. Distilled water was poured into a volumetric flask up to the mark of 50 mL and flask was further subjected to sonication. Drug was extracted from 50 mL solutions prepared previously using bath sonicator, with sonication time of 2 minutes. The actual drug content was determined using a UV–visible spectrophotometer (Shimadzu UV-1700 spectrophotometer, Japan) at 277 nm and the drug concentration was determined using constructed standard calibration curve, covering the drug solution concentration from 5.0-50.0 µg/mL.

*Radiolabelling the tablets with Technetium-99 (* ^99m^ *Tc)*

Tablets were radiolabelled by drilling a small hole (1 mm) through the center of the tablet with an electric driller and introducing a radioactive solution (15). Technetium (^99m^Tc) was chosen for radiolabelling of the tablets because of its short half-life of 6 h along with optimum electron emission. Diethylenetriamine-pentaacetic acid (DTPA) was used to provide the necessary ^99m^Tc species for radiolabelling (16). In a vial, 100 µL of ^99m^Tc-labeled DTPA was taken and 1 mg of stannous chloride dihydrate (1 mg/mL in 10% acetic acid) was added. The pH was adjusted to 7.5 using 0.5 M sodium bicarbonate solution and mixture was stirred for 5 minutes. Afterwards 4–7 µL of aqueous solutions of ^99m^Tc-DTPA of known radioactivity (100 MBq) was instilled in to the drilled holes, avoiding contact with the surface of the tablets. The radioactive solution was left to diffuse for 15 minutes and then dried to assure uniform dispersion of radioactive material within the matrix. Ethanolic solutions of graft copolymers and lactose (9:1) were used to fully seal the drilled holes. The strength of radioactive label (MBq) was determined by CAPINTEC CRC-15R detector (Pittsburgh, U.S.A).

*Stability of radiolabelled tablets*

Stability of ^99m^Tc-labeled graft copolymer matrix tablets (F3 & F4) was tested in standard buffer solutions of pH 1.2, 6.8 and 7.4 in order to confirm that the activity would not leach out from the tablets during transit time of the formulation through GI tract. Dissolution tests for the release of the radioactive material was performed by placing radiolabelled tablets in USP type II paddle type dissolution apparatus containing different standard buffers solutions (900 mL), and it was operated at rotation speed of 50 rpm at 37 ± 0.5 °C for 6 h. At each 30 min interval, 5 mL of sample was withdrawn from dissolution vessel and withdrawn volume of dissolution media was replaced with fresh buffer solution. At the end of the dissolution testing, tablets were recovered from the dissolution medium and were further blotted using tissue paper. Activity of the test solutions was determined using a CRC-15R detector. The amount of radioactive strength remained in the tablets was estimated by dissolution testing. 

*In-vitro drug release studies*

Drug release studies were conducted on ^99m^Tc-DTPA labeled tablets and compared with unlabelled tablets to determine whether the labeling process affects the kinetics of drug release from the tablets. Drug release study was carried out using USP dissolution type II apparatus with paddle rotating at 50 rpm**. **Dissolution study was performed in simulated gastric fluid (0.1N HCl) and in simulated intestinal fluid (PBS pH 6.8) for 14 h**. **The temperature of dissolution medium (900 mL) was maintained at 37 ± 0.5ºC throughout the study. Tablets were placed in different baskets. At a predetermined time intervals of 0, 1, 2, 3, 4, 5, 6, 8, 10, 12 and 14 h, 5 mL sample was withdrawn using a syringe fitted with 0.45 μm filter, and withdrawn volume was replaced with the fresh medium to maintain sink condition**. **Samples were analyzed at 277 nm, using UV-visible spectrophotometer. The corresponding cumulative percent of drug released was determined using the constructed standard calibration curve at this wavelength covering the range for the assay. Mean release of three tablets was used to evaluate the drug release for each of the formulations (17).

*Kinetics of drug release*

To study the mechanism of drug release from the optimized formulation of matrix tablets, *in-vitro* release profiles were correlated with various kinetic models like zero order (cumulative amount of drug released vs. time), first order (log cumulative percentage of drug remaining vs. time), Higuchi model (cumulative percentage of drug released vs. square root of time) and Korsmeyer-Peppas (log cumulative percentage of drug released vs. log time) release equations.

*In-vivo Pharmacokinetic studies on rabbits*

*Bio-analytical method development*

A high performance liquid chromatography system of Adept series CECIL CE 4201 with UV/Visible detector was used for analysis. The data were recorded by using the software “Power Stream”. The column used for separation was octadecylsilane (C_18_) with length 250 mm, internal diameter 4.6 mm (Phenomenex) and particle size 5 μm. Chloramphenicol was used as an internal standard**. **In 0.5 mL of plasma sample, 20 μL of internal standard (100 μg/mL) was added. Drug was extracted from plasma using 5 mL methanol**. **Mobile phase used for analysis contains acetonitrile, methanol and water in the ratio of 60:20:20 (v/v), and pH adjusted to 2.8 with orthophosphoric acid, with a flow rate of 0.5 mL/min. Sample of 20 μL was injected manually and chromatogram was recorded. Quantification of salbutamol sulphate was performed by plotting peak area ratio of SS to the internal standard as a function of its concentration.

*Assay method validation*

The developed method was validated following bioanalytical guidelines (18). Bioanalytical method validation required the determination of selectivity, linearity, LOD, LOQ, accuracy, recovery and precision respectively.

*Animal experimental protocol for pharmacokinetic study*

*In-vivo* studies were performed as per the guidelines of the Council for the Purpose of Control and Supervision of Experiments on Animals, Ministry of Social Justice and Empowerment, Government of India. Pharmacokinetic parameters of graft copolymer formulations (F3 and F4) were compared with tablets prepared by native starch (F1), acetylated starch (F2), HPMC K100M (F5) and commercial sustained release tablets (Asthalin SA-8 mg).

Male albino rabbits weighing 2.5-3.0 kg were randomly selected for the bioavailability study. The animals were subdivided into six groups and each group was comprised of three rabbits. Each group received of the tested formulas namely F1, F2, F3, F4, F5 and Asthalin SA-8 mg. The animals were fasted over night before tablet administration and during the experiment all rabbits had free access to water. Tablets were put behind the tongue to avoid their destruction due to biting. Blood samples (about 1 mL from each animal) were collected from the orbital sinus, before dosing (zero time) and at different intervals after dosing *viz*. 1, 2, 3, 4, 5, 6, 8, 10, 12, 14, 16, 18, 20, 22, and 24 h. Samples were collected in micro centrifuge tubes containing 50 µL of 10% w/w of disodium EDTA as anticoagulant. The collected samples were immediately centrifuged at 10000 rpm for 10 minutes and plasma were separated and stored at –20 °C.

*Statistical analysis*

Statistical significance was determined by one way ANOVA (Analysis of variance) (Graph Pad Instat software v 3.06, CA, USA). Significant differences between formulations were analyzed using *student newmann-keuls *multiple comparison test and p-values of <0.05 were considered to be statistically significant. 

*In-vivo imaging and animal study protocol for gamma scintigraphy*

The whole experiment was approved by “Institutional Animal Ethical Committee” according to the rules of “Committee for the Purpose of Control and Supervision of Experiments on Animals” (CPCEA, Registration number Dean/10-11/57) for the care and use of laboratory animals were strictly followed throughout the experiment. Gastrointestinal transit of tablets was obtained by imaging studies using ^99m^Tc as a radioactivity marker. Male albino rabbits weighing 2.5-3.0 Kg were randomly selected for gamma scintigraphic study. Rabbits were divided into 3 groups, having 3 animals in each and were fasted for 12 h prior to the gamma scintigraphic study. Group-I received radiolabelled starch matrix tablet (F1), group-II & III received graft copolymer containing matrix tablets F3 and F4 respectively; followed by sufficient amount of drinking water. Animals were anaesthetized with diethyl ether and serial scintigraphic examination was done at 0.5, 1, 1.5, 2, 3, 5, 7 and 10 h to visualize the transit of tablets in GIT, using a large field view gamma camera equipped with a high-resolution, parallel-hole collimator. The 140 keV gamma rays emitted by ^99m^Tc were imaged. Gamma images were recorded using an online computer system and analyzed to determine the distribution of radioactivity in the stomach, intestine and colonic region.

## Results and Discussion

*Preformulation study*

*Drug–excipient compatibility study by FT-IR spectroscopy*

**Figure 1:**
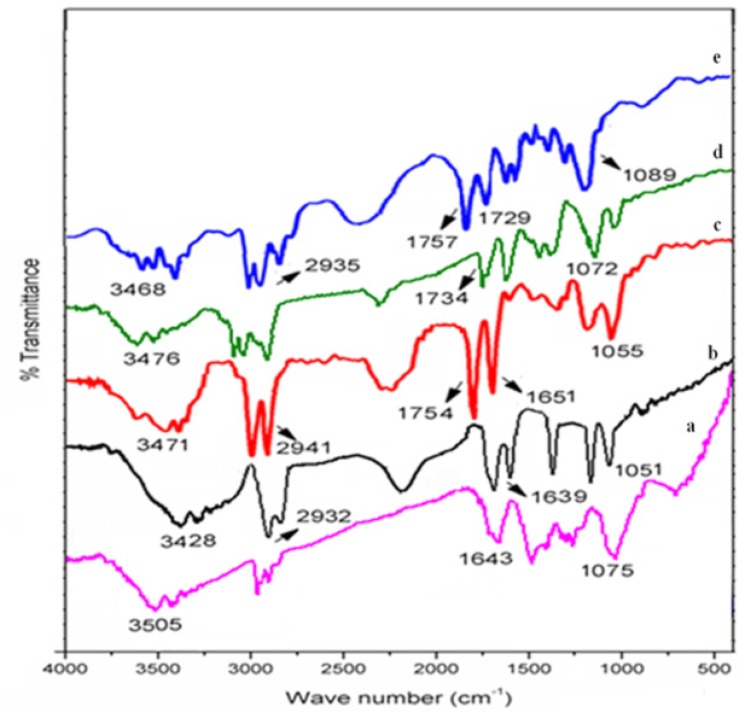
FTIR spectra of (a) salbutamol sulphate (b) starch and SS blend (c) acetylated starch and SS blend (d) St-g-PMMA and SS blend (e) Ast-g-PMMA and SS blend.

The IR spectrum of salbutamol sulphate showed sharp peaks at 1075 cm^-1^ corresponding to C-O stretching and at 1643 cm^-1^ for O-H bending. Broad peaks appeared at 3200-3600 cm^-1^ corresponding to OH and NH stretching (Figure 1a). In drug-polymer blends of various formulations, some identical peaks were also present which confirmed the compatibility between drug and polymers (Figure 1b-e).

*Angle of repose*

Prior to compression, trials blends (F1-F5) were evaluated for their flow property. From the values of angle of repose, it is evident that blends having graft copolymers showed better flow properties than that of blends having acetylated starch and native starch (Table 2). Trial batches were easily compressed on a single punch manual compression machine and the resulting tablets were evaluated using pharmacopoeial tests.

**Table 2:** Angle of repose as an indicator of powder flow property (All values are expressed as mean ± SD, n=3).

**Formulation code**	**Angle of repose**	**Type of flow**
F1	32.34° ± 0.11	Passable
F2	30.41° ± 0.23	Passable
F3	25.73° ± 0.17	Good
F4	23.45° ± 0.19	Good
F5	19.11° ± 0.20	Excellent

*Standard physical test of tablets*

Tablets prepared (F1–F5) with mentioned array of excipients had thickness varying from 3.19-3.41 mm (Table 3). Drug content was found to be uniform among different batches of the tablets and ranged from 100.98 to 104.21%. Hardness and percentage friability of the tablets of all the batches ranged from 3.27 to 4.01 Kg/cm^2^ and 0.53 to 0.84%, respectively. Tablets with all aforesaid composition passed USP criteria for friability (<1.00% w/w). Outcomes from friability assessment revealed excellent mechanical strength of the tablets. The weight variation of individual tablet was found to be within ± 5% w/w of average weight of tablets, which affirmed that all the tablets have passed the USP weight variation test.

**Table 3:** Physical properties of formulated salbutamol matrix tablets using graft copolymers and HPMC K100M as release retardants. (All values are expressed as mean ± SD, n=3).

**Formulation code**	**Weight deviation (mg)**	**Hardness (Kg/cm** ^2^ **)**	**Friability (%)**	**Thickness, mm**	**% Drug content**
F1	204 ±2.1	3.27 ±0.57	0.75 ±0.24	3.24 ±0.22	102.47 ±2.6
F2	206 ±2.1	3.35 ±0.87	0.53 ±0.21	3.26 ±0.52	100.98 ±2.3
F3	203 ±1.9	3.49 ±0.92	0.69 ±0.17	3.37 ±0.47	101.57 ±2.8
F4	205 ± 2.3	4.01 ± 0.53	0.77 ± 0.29	3.19 ± 0.38	104.21 ± 2.9
F5	204 ± 2.7	3.76 ± 0.73	0.84 ± 0.19	3.41 ± 0.29	103.61 ± 3.1

*In-vitro drug release studies*

**Figure 2:**
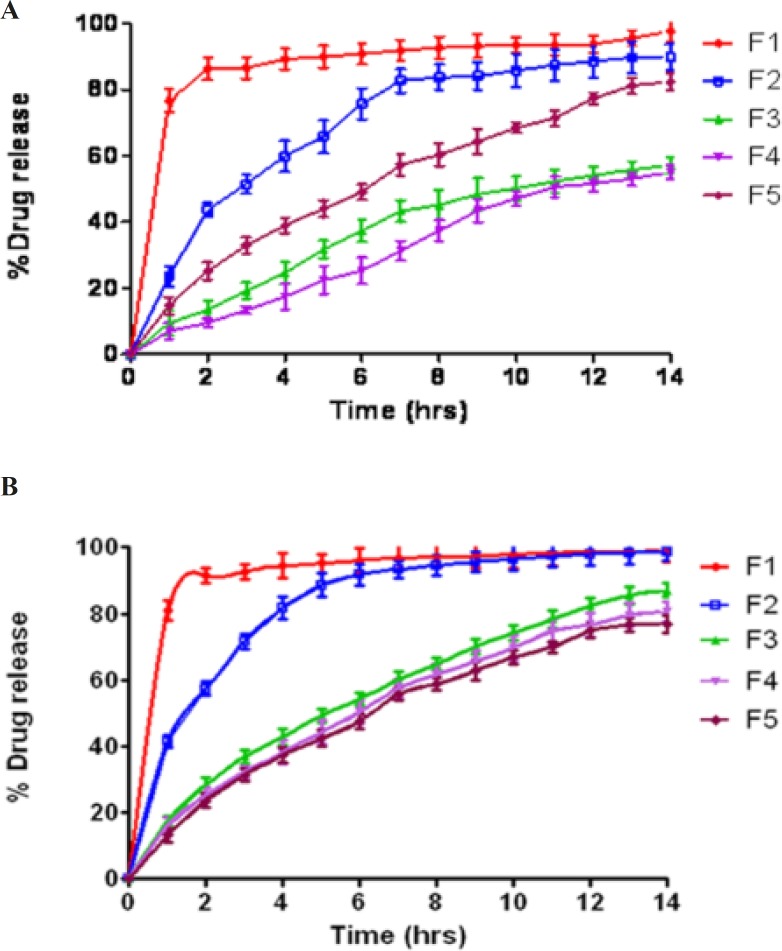
Release profiles of model drug SS in (A) SGF and (B) SIF at 37 ºC, with starch (F1), acetylated starch (F2), St-g-PMMA (F3), Ast-g-PMMA (F4) and HPMC K100M (F5) as carriers.

The *in-vitro* release profiles of salbutamol sulphate matrix tablets in simulated gastric fluid (SGF) and simulated intestinal fluid (SIF) are presented in Figure 2. The studies were performed for 14 h and a higher percentage of drug release was found in matrices containing native starch (F1) compared with the ones having acetylated starch (F2) and graft copolymers (F3 & F4) in both the SIF and SGF. The release of drug was prolonged up to 14 h for matrices containing graft copolymers (F3 & F4) and up to 6 h for matrices containing acetylated starch (F2) whereas in case of starch >80% drug was released within 1 h. Prolonged and sustained release behavior of graft copolymer matrices could be attributed from the superior binding properties of the polymers, while fast drug release behavior of starch containing tablet was attributed from the burst release of the drug due to tablet breaking after immersing in dissolution media.

The drug release rate of graft copolymers was greater in SIF than in SGF perhaps due to enhanced swellability of carrier in SIF, because of ionization of carboxyl groups. The swelling is dependent on the intermolecular interaction between the polymer components of grafted copolymer. At lower pH, the carboxyl group of the grafted methyl methacrylate chain remains almost in unionized state might be due to involvement of hydrogen bond formation with hydroxyl group of starch, which made the polymer segments rigid and thereby retarded the water uptake and decreased the extent of swellability.Whereas at higher pH the carboxyl groups get ionized, which reduced the starch–acrylic interaction by decreasing H-bonding; the repulsion among similarly charged –COO^– ^group increased chain relaxation; and ionic nature of chains facilitated the water uptake resulting increase in the extent of swelling at higher pH. While using starch as a carrier, 80–85% of drug was released in SIF during the initial 1h and this trend decreased in case of acetylated starch to 41% of drug release. Further in case of St-g-PMMA and Ast-g-PMMA as a carrier, drug release reduced to 17.5% and 16.1% respectively. In SGF, a remarkable decrease was observed in graft copolymer matrices *i.e.* 9.5% in case of St-g-PMMA in initial 1h and decreased to 7.01% in case of Ast-g-PMMA. Comparison of drug release pattern from graft copolymers matrices (F3 and F4) with HPMC K100M containing tablets in SIF revealed that matrix tablets (F3 and F4) released 82.45% and 76.71% of drug respectively within 12 h while HPMC K100M tablets released 74.8% of drug content within 12 h. So, retardation of drug release rate of formulations having graft copolymers (F3 & F4) was almost equivalent to commercially used controlled release polymer *i.e*. HPMC K100M which supported their application as potential excipients in controlled drug delivery system.

**Figure 3:**
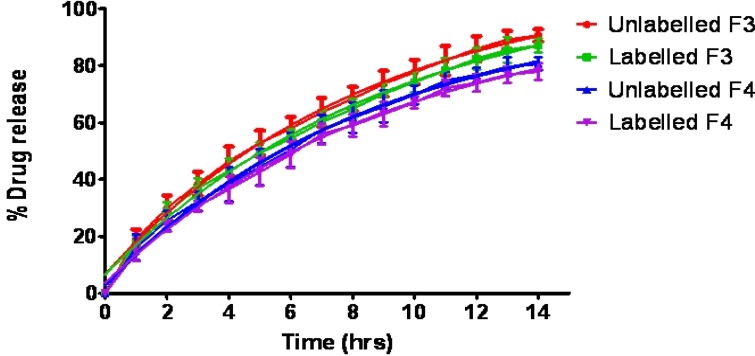
Percentage drug released with time from radiolabelled and unlabelled graft copolymer matrix tablets.

The *in-vitro* dissolution profiles of salbutamol sulphate from radiolabelled and unradiolabelled graft copolymer matrix tablets (F3 & F4) in the simulated intestinal fluid (pH 6.8) were investigated, in order to determine the possible effect of the labelling process on the kinetics of drug release from the matrix tablets. By analyzing the results compiled in Figure 3, it was concluded that ^99m^Tc did not intervene with the drug release. In fact, it is distinctly mentioned that the dissolution profiles of labelled and unlabelled tablets were basically super imposable, suggesting that this technique did not affect the drug release rate. Actually, the cumulative amount of drug released from the tablets after 14 h was 90.64 ± 2.19% (unlabelled), and 87.11 ± 2.48% (labelled), for formulation F3; 80.67 ± 2.22% (unlabelled) and 77.96 ± 2.88% (labelled) for formulation F4.

*Kinetics and mechanism of drug release*

The drug release mechanism was found by fitting the *in-vitro* dissolution data into various kinetic models and the values of release exponent (n_p_), kinetic constant (K) and regression coefficient were determined, as indicated in Table 4. Zero order, first order, Higuchi, and Korsmeyer-Peppas are the major models to key out the drug release from sustained release formulations and criteria of selecting the most appropriate model was based on the regression coefficient. Starch and acetylated starch tablets (F1 & F2) showed first order release, with regression value of 0.858 and 0.979 respectively.

*In-vitro* release proﬁle of graft copolymers matrix formulations (F3 & F4) and HPMC K100M tablets (F5) were best expressed by the Higuchi model, as the plots showed high linearity with regression value of 0.995, 0.991, and 0.996 respectively. However, Higuchi’s equation failed to explain the influence of swelling of the matrix upon hydration and gradual erosion of the matrix. Therefore, the dissolution data were fitted into the well-known exponential Korsmeyer-peppas equation; here the value of release exponent (n_P_) explains the release mechanism of the drug from the matrix system. Observed ‘n_P_’ values for release profiles of formulation F3, F4 and F5 were in between 0.50 to 0.89 indicating anomalous release behavior coupled with diffusion and erosion. The release exponents for formulation F1 was found less than 0.5 indicating Quasi fickian diffusion mechanism for the drug release.

**Table 4:** Comparative release kinetics parameter of all the batches of controlled release tablets in SIF.

**Formulation code**	**Release kinetic parameters**							
	**Zero order**		**First order**		**Higuchi**		**korsemeyer-peppas**	
	r^2^	K_0_	r^2^	K_1_	r^2^	K_H_	r^2^	n_P_
F1	0.324	3.18	0.858	0.109	0.576	17.92	0.788	0.33
F2	0.646	5.25	0.979	0.129	0.864	25.67	0.830	0.49
F3	0.949	5.71	0.991	0.061	0.995	24.68	0.929	0.63
F4	0.951	5.42	0.989	0.051	0.991	23.38	0.988	0.67
F5	0.937	5.36	0.993	0.052	0.996	23.33	0.992	0.60

*Bio-analytical method validation*

*Selectivity*

**Figure 4:**
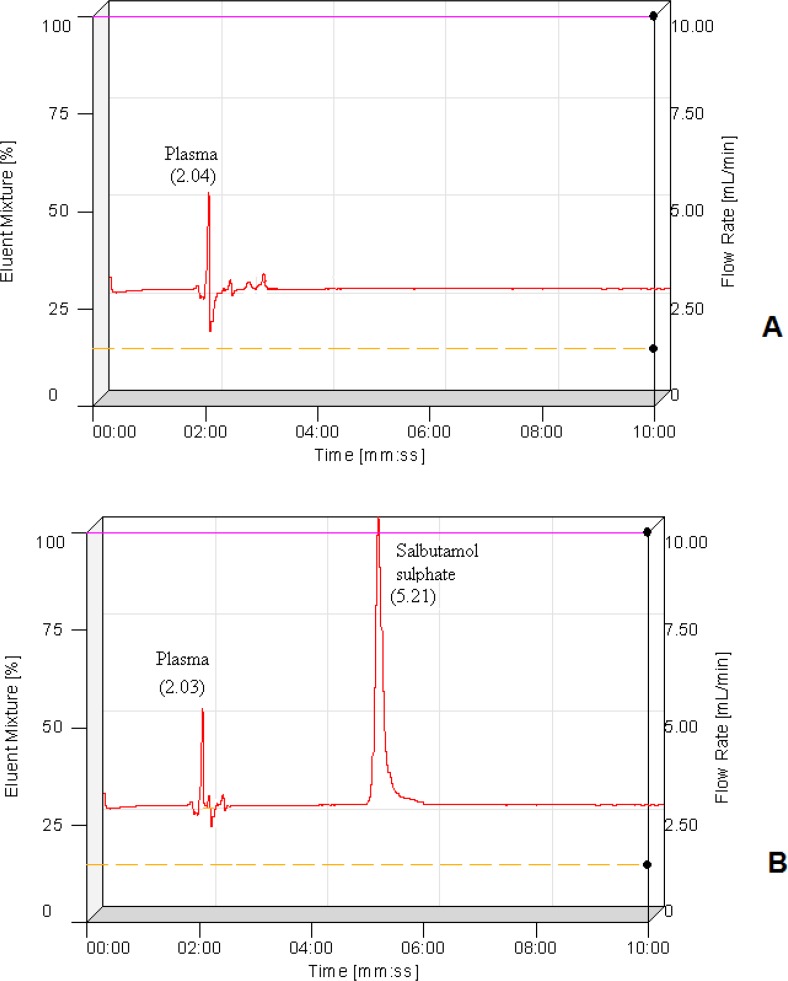
HPLC chromatograms of A) blank plasma and B) salbutamol sulphate extracted from plasma.

The chromatograms of blank plasma and a spiked plasma sample are shown in Figure 4 (A & B). With the chromatograms it is clear that retention times of blank plasma (2.04 min) and salbutamol sulphate (5.21 min) are quite different means plasma components are not showing interference with the drug elution.

*Limit of detection (LOD) and Limit of quantification (LOQ)*

LOD and LOQ for *salbutamol sulphate* were 33.97 ng/mL and 101.91 ng/mL, respectively. 

*Linearity*

Standard calibration curve in rabbit plasma was found to be linear at concentrations ranging from 100 to 1,200 ng/mL (Y = 0.0318x + 1.4027) with correlation coefficient of 0.9968.

*Accuracy*

Recovery studies were performed to validate the accuracy of developed method. To the preanalysed sample solution, a definite concentration of standard drug was added and then its recovery was analyzed. The method was found to be accurate if % recovery is 100 ± 15% and % CV is <15%. The % recovery of drug in plasma found in the range of 89.59 -92.41% and coefficient of variation was 2.21-3.81. Since the % recovery and % CV is within the range, it shows accuracy of method. 

*Precision*

Precision of the method was assessed by intra-day and inter-day analysis of six replicates for each concentration at 3 different concentration levels (100, 600, and 1200 ng/mL, respectively). The coefficient of variation (CVs) at each concentration level was expressed as precision. The method proved to be precise because the % CV for is not more than 5.61% at three different concentration levels. 

*In-vivo pharmacokinetics*

**Figure 5:**
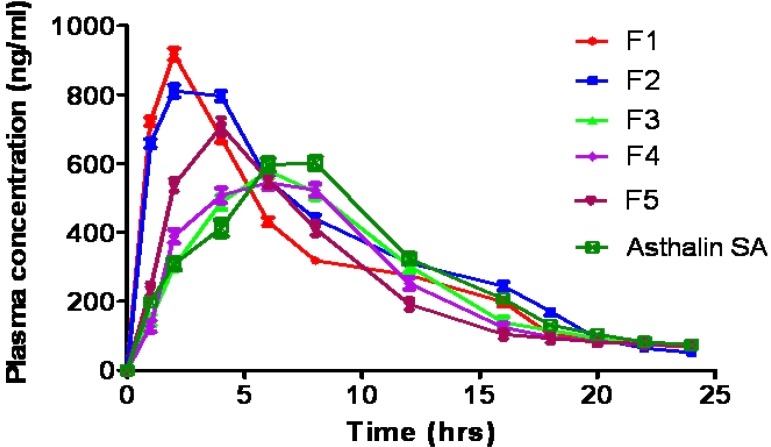
Comparative *in-vivo* peak plasma concentration of starch (F1), acetylated starch (F2) matrix tablets, graft copolymer matrix tablets (F3 and F4), HPMC K100M matrix tablets (F5) and Asthalin SA.

The results of plasma drug concentration at different time intervals, after administration of tablets of formulation F1-F5 and Asthalin SA 8 mg tablets to rabbits, are shown in Table 5. SS was found and quantified in plasma by using HPLC method and mean plasma concentration curve of formulated tablets and commercial tablets are shown in Figure 5. The plasma kinetic data were assessed with Kinetica ® software (version 5). The maximum SS concentration in serum (C_max_) and corresponding peak time (t_max_) were determined by assessing the individual serum drug concentration-time profiles. The elimination rate constants (Ke) were obtained from least square fitted terminal log-linear portion of the serum concentration-time profile. The elimination half life (t_1/2_) was calculated as 0.693/Ke. The area under the plasma concentration [AUC]_ 0–24 _vs time curve was determined by linear trapezoidal rule until the last measurement point.

The C_max_ of graft copolymer matrices F3 (578.9 ng/mL) and F4 (546.7 ng/mL) was significantly (p<0.05) lower than starch matrix F1 (924.1 ng/mL) and acetylated starch matrix tablets (812.4 ng/mL). Decrement in C_max_ value of the controlled release tablets of graft copolymers F3 and F4 indicated their sustained and prolonged effect. The t_max_ of graft copolymer matrices F3 (6 h) and F4 (7.5 h) was significantly (p<0.05) higher than starch matrix F1 (1.5 h) and acetylated starch matrix tablets (2.5 h), which indicated slow absorption rate from the graft copolymer tablets due to extended release effect of hydrophobic polymer. Different elimination rate constants of various formulations may be due to difference in the metabolism pathway of polymer matrices. The elimination half life (t½) of the F3 (12.15 h) and F4 (13.07 h) was more than F1 (5.45 h) and F2 (6.72 h), which confirmed the prolonged availability of SS in graft copolymer matrix tablets. In all formulations, large difference was observed between plasma half life and t_max _which reflects significant difference between absorption and elimination time of the drug.

However there was no significant difference in the pharmacokinetic parameters (t_max_, C_max_, AUC, Ke, and t_1/2_) of the graft copolymers matrices, HPMC K100M matrix tablets and commercial tablets. The results revealed that the graft copolymer matrices provided comparable sustained and prolonged effect as compared to HPMC K100M matrix tablets and marketed product (Asthalin SA 8 mg tablets). Therefore graft copolymers can be used as potential excipients in controlled drug delivery system.

**Table 5:** Pharmacokinetic parameters of formulated (F1, F2, F3, F4 & F5) and marketed tablets (Asthalin SA) of salbutamol sulphate in rabbits (n=3).

**Pharmacokinetic parameters**	**F1**	**F2**	**F3**	**F4**	**F5**	**Asthalin SA**
Maximum plasma concentration, C_max_ (ng/ml)	924.1 ± 10.1	812.4 ± 7.9	578.9 ± 9.1	546.7 ± 8.9	598.1± 10.7	601.9 ± 6.3
Time required to reach maximum plasma concentration, t_max_ (h)	1.5 ± 0.23	2.5 ± 0.47	6.0 ± 1.3	7.5 ± 1.2	6.2 ± 1.1	7.1± 0.75
Area under curve at 24h, AUC (ng-h/mL)	5261.19 ± 5.1	4979.52 ± 5.1	6460.12 ± 8.3	6679.48 ± 7.5	6276.34 ± 6.8	6592.28 ± 6.8
Elimination rate constant, Ke (h^-1^)	0.127 ± 0.017	0.103 ± 0.019	0.057 ± 0.011	0.053 ± 0.011	0.049 ± 0.012	0.059 ± 0.014
Plasma half life, t_1/2_ (h)	5.45 ± 0.59	6.72 ± 0.31	12.15 ± 0.91	13.07 ± 1.21	14.14 ± 1.09	14.43 ± 0.87

*Gamma scintigraphy studies*

*Stability of radiolabelled tablets*

The activity released from ^99m^Tc labelled-drug loaded graft copolymer matrix tablets at different pH for study period of 6 h are shown in Table 6. More than 73% of the estimated radioactivity was retained in the tablet even after 6 h of dissolution in different media. In acidic media (pH 1.2), percent radioactivity release was least which indicates stability of graft copolymers was higher in acidic media. Thus, the labeling procedure was appropriate because there was no sudden release of radioactivity into the dissolution media.

**Table 6:** Stability data of 99mTc labelled-drug loaded graft copolymer matrix tablets

pH	% radioactivity released in dissolution medium after 6 h	
	**F3**	**F4**
**1.2**	**14.56 ± 2.67**	**12.19 ± 1.07**
**6.8**	**24.91 ± 2.91**	**21.56 ± 1.74**
**7.4**	**26.59 ± 2.91**	**25.71 ± 2.03**

*In-vivo imaging study on rabbits*

**Figure 6:**
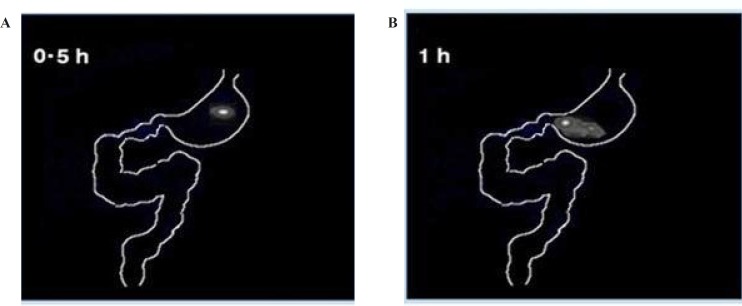
Gamma scintigraphy study of starch matrix tablets (F1) on rabbits at time point (A) 0.5 h and (B) 1 h.

**Figure 7:**
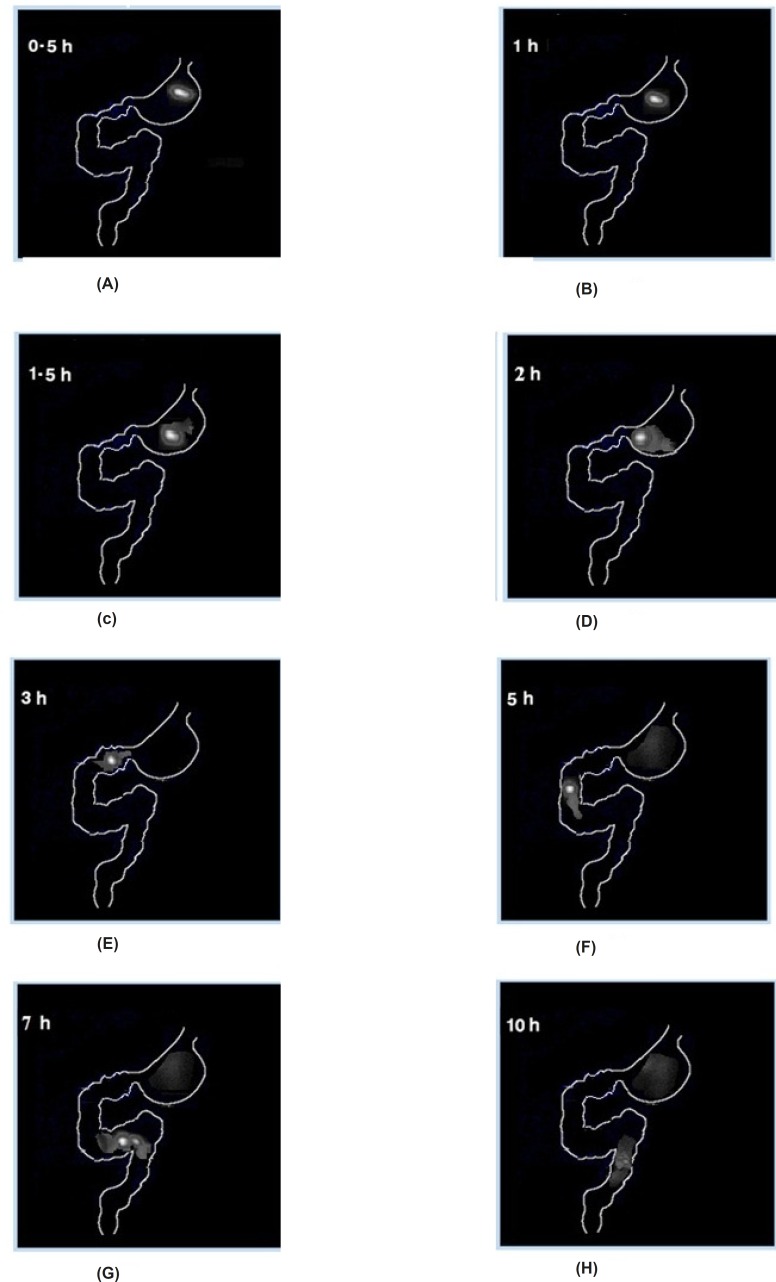
Gamma scintigraphic images of graft copolymer matrix tablets (St-g-PMMA, F3) on rabbits at time point (A) 0.5 h, (B) 1 h, (C) 1.5 h, (D) 2 h, (E) 3 h, (F) 5 h, (G) 7 h and (H) 10 h.

The gamma scintigraphic study was conducted in order to assess gastro-retentive behavior and overall gastrointestinal transit of the two optimized graft copolymer matrix formulations (F3 & F4) in rabbits in comparison with starch matrix tablets (F1). Scintigraphic images of starch matrix tablets (F1) clearly showed attenuation of the radioactivity within 1 h Figure 6 (A & B). This suggests that in the presence of gastric fluid, starch matrix tablets got dissolved completely and behaved as an immediate release dosage form. The graft copolymers matrix tablets (F3 & F4) were found to maintain their matrix integrity till 2 h in the gastric region indicating the absence of gastric fluid influence on the graft copolymer matrices, scintigraphhic images of St-g-PMMA matrix tablets were shown in Figure 7 (A, B, C & D). In the formulation (F3 & F4), tablets were observed to have migrated into the duodenum where the integrity of formulation was still maintained (Figure 7E). Further, in the 5^th ^h, tablet reached to the jejunum region where the integrity of the formulation was slightly affected (Figure 7F). This indicates pH responsive behavior of graft copolymer matrices and suggested that they are not only valuable to retain the dosage form in the stomach for more than two hours but can likewise protect the drug in an alkali environment for few h. The image of 7^th ^h depicted that, in the proximal colon region of gastro intestinal tract (GIT), tablet starts dispersing corresponding to attenuate radioactivity (Figure 7G) and images of 10^th ^h showed complete disintegration of the dosage form (Figure 7H). The results related to the gastric transit and disintegration of the tablets in the intestinal lumen is in full accord with the data mentioned in the pharmacokinetics and *in-vitro* drug release studies.

## Conclusions

From the present work, it can concluded that tablets prepared by salbutamol sulfate as a model drug and graft copolymer matrices as release retarding agent are capable of giving up drugs for a longer duration and their drug release characteristics are comparable with a controlled release matrix of HPMC K100M. Results of preformulation study revealed that graft copolymerization improved the flow property of native starch. Matrix tablets prepared by graft copolymers and HPMC K100M gave slow release of the drug up to14 h. The *in-vitro* drug release rate was greater in SIF as compared to SGF which showed pH sensitivity and site-specific drug delivery of graft copolymer matrices. SIF facilitates ionization of the carboxyl groups of the grafted methyl methacrylate chain which enhances the repulsion between similarly charged COO^– ^group rendering to chain relaxation; and ionic nature of chains facilitates water uptake, with the overall increase in the drug release in the dissolution medium of higher pH. In graft copolymers matrices, drug release was controlled by diffusion and erosion. The HPLC method used for *in-vivo* pharmacokinetic study has been validated according to U.S. Food and Drug Administration guidelines for bioanalytical methods and found to be linear, accurate and precise both in upper and lower concentration range with acceptable error and % CV values. *In-vivo* pharmacokinetic study revealed that in case of graft copolymer matrices (F3 & F4), value of t_max _was higher and C_max_ was lower than that of starch and acetylated starch matrix tablets (F1& F2). It reflects that graft copolymerization markedly influence the capacity of starch/acetylated starch in modulating drug release. Pharmacokinetic parameters of graft copolymers matrices (F3 & F4), HPMC K100M matrix tablets (F5) and marketed product were almost comparable, indicating ability of graft copolymers as promising vehicles for sustained release formulations in oral drug delivery system.

The Gamma scintigraphic images were showed the controlled release behavior of graft copolymer formulation which revealed their suitability for intestinal drug delivery system. Results of *in-vitro *and *in-vivo* studies were well correlated and confirm the ability for graft copolymer formulations to release the drug only after a desired period of time in a controlled manner.
